# Biomedical Informatics and Computational Biology for High-Throughput Data Analysis

**DOI:** 10.1155/2014/398181

**Published:** 2014-02-12

**Authors:** Bairong Shen, Jian Ma, Jiajun Wang, Junbai Wang

**Affiliations:** ^1^Center for Systems Biology, Soochow University, P.O. Box 206, Suzhou 215006, China; ^2^Department of Bioengineering, University of Illinois at Urbana-Champaign, Urbana, IL 61801, USA; ^3^School of Electronics and Information Engineering, Soochow University, Suzhou 215006, China; ^4^Department of Pathology, The Norwegian Radium Hospital, Oslo University Hospital, Montebello, 0310 Oslo, Norway

The last few years have witnessed the emergence of several high-throughput (HTP) platforms that are based on the evolution of omic science through transcriptomics, proteomics, and metabolomics. With the huge data accumulation, the informatics and computational methods have become essential to understand the complexity of biomedical data. We lunched the special issue to address the demand for statistical models, biological omic data analyses, and meta-analysis of biomarker for complex diseases.

Statistical models are always important for the understanding of complex data; in this issue, Y. Liang et al. proposed a new adaptive L1/2 shooting regularization method for variable selection based on the Cox's proportional hazards mode. Simulation and the real gene expression dataset analysis showed that the method is more accurate for variable selection than Lasso and adaptive Lasso methods. L. Tian et al. employed a nonlinear model to analyze time course gene expression data. They firstly developed a method for estimating the parameters in the nonlinear model and then utilized the model to perform the significance analysis of individually differentially expressed genes and clustering analysis of a set of gene expression profiles. The simulation and real-life biological data analyses showed that their methods outperform some existing methods.

For the application of bioinformatics tools, Z. E et al. analyzed the gene expression in rice leaf blades at different temperatures. They analyzed the next-generation sequencing (NGS) datasets and characterized the transcription profiles of the samples in rice seedling leaf blades at 25°C and 30°C. Gene Ontology (GO) and Kyoto Encyclopedia of Genes and Genomes (KEGG) databases were applied to study the effect of the temperature. Finally, they observed that temperature markedly regulates several superfamilies of transcription factors, including bZIP, MYB, and WRKY. Chronobiology is a field of biology that studies biological rhythms. R. Lopes et al. reviewed the bioinformatics or computational systems biology methods, tools, and databases which are very helpful to the understanding of the patterns and biological rhythms found in living organisms and the review will benefit the community for the application of bioinformatics in their future researches.

For the understanding of protein structure and structure-function relationship, two papers are selected in this issue. M. Shambhu et al. studied the surface accessibility of hydrophobic residues by considering their conservation score and knowledge of flanking regions. The accuracy of prediction is therefore improved. In the other work, D. Lu et al. applied molecular dynamics to investigate the effects of X-linked agammaglobulinaemia associated with amino acid mutations in pleckstrin homology (PH) domain on the binding of the domain with inositol 1,3,4,5-tetrakisphosphate (Ins(1,3,4,5)P4). The mutations were then classified as “functional mutations”, and “folding mutations.” The result provided new insights into the biological function of the Btk-PH domain and related mutation-causing diseases.

Finally, two works in this special issue are medical studies. The first is the work by B. Lv et al. They analyzed the 347 studies for the diagnostic value of tumor necrosis factor-*α* (TNF-*α*) and found that the northern hemisphere group in the TNF-*α* test has higher sensitivity and specificity than those of southern hemisphere group. The second medical study is an association study. Z. Liu et al. reported the significantly association of NCK2 with opiates addiction in African-origin men by a comprehensive analysis of a dataset from the Study of Addiction: Genetics and Environment (SAGE). They employed both SNP and gene based methods of analysis and identified a strong and significant association between a SNP in the NCK2 gene on chromosome 2 with opiates addiction in African-origin men.

*Bairong  Shen* *Bairong  Shen* *Jian  Ma* *Jian  Ma* *Jiajun  Wang* *Jiajun  Wang* *Junbai  Wang* *Junbai  Wang*

